# An Account of the Medicinal Plants Growing in Jamaica

**Published:** 1787

**Authors:** William Wright

**Affiliations:** the Royal College of Physicians and Royal Society of Edinburgh


### LONDON MEDICAL JOURNAL,

FOR THE YEAR 1787,

### PART THE THIRD.

I. *An Account of the Medicinal Plants growing in Jamaica. By* William Wright, *M. D. F. R. S. and of the Royal College of Physicians and Royal Society of Edinburgh. Communicated in a Letter to Sir* Joseph Banks, *Bart. P. R. S. and by him to Dr.* Simmons.

*To Sir* JOSEPH BANKS, *Bart. P. R. S.*

SIR,

AT the request of the late Dr. Fothergill and Dr. Solander, I drew up an account of the officinal plants growing in Jamaica for the Medical Society of London; but the death of those valuable friends, and the dissolution of that Society, have occasioned it to remain unpublished. Having now revised this paper, and added thereto a considerable number of observations and facts, I take the liberty, Sir, of presenting it to you as a testimony of my respect, and, if it meets with your approbation, I request the favour of you to transmit it to Dr. Simmons, to be inserted in the London Medical Journal.

I have the honour to be, Sir,
Your most obedient and very
Humble Servant,

WILLIAM WRIGHT.

*Edinburgh,*
May 27th, 1787.

### INTRODUCTION.

I BEG leave to observe that the following descriptions of plants were made on the spot, and that the medical remarks are the result of careful observation and experience in the practice of physic for many years in Jamaica.

I flatter myself that I shall be found to have made discoveries, new and important, which have escaped the notice of Sloane, Jacquin, and Browne, and that what I have written will throw some light on the history of the materia medica. If men of abilities and observation would contribute thus to the public stock, we might hope that the history of foreign drugs would soon be made more perfect.

1. Aloe perfoliata. — *Hepatic Aloes.* — *Caballine Aloes.* — *Barbadoes Aloes.*

This is a common plant in all the Weſt-India iſlands. It is known by the name of *ſempervivum*, and is cultivated particularly in Barbadoes.

This plant flowers in June, but bears no ſeed: the young ſhoots from the roots ſerve to propagate it.

Hepatic aloes is obtained in the following manner:—The plant is pulled up by the roots, and carefully cleanſed from the earth or other impurities. It is then ſliced and cut in pieces into ſmall hand baſkets or nets. Theſe nets or baſkets are put into large iron boilers with water, and boiled for ten minutes, when they are taken out, and freſh parcels ſupplied till the liquor is ſtrong and black.

At this period the liquor is thrown through a ſtrainer into a deep vat, narrow at bottom, to cool, and to depoſit its fæculent parts. Next day the clear liquor is drawn off by a cock, and again committed to the large iron veſſel. At firſt it is boiled briſkly, but towards the end the evaporation is ſlow, and requires conſtantly ſtirring to prevent burning. When it becomes of the conſiſtence of honey, it is

 poured poured into gourds, or calabafhes, for fale. This hardens by age.

2. ALOE SPICATA. — *Succotrine Aloes.*

About twelve years ago Dr. Fothergill fent this plant to Jamaica for the botanic garden there; but by the removal of the garden to a diftant part of the country, this and feveral other valuable plants were loft. Had it been propagated, it would have proved a valuable acquifition to the ifland. The gum may be prepared as above.

3. AMOMUM ZINZIBER. — *Ginger.*

There are two forts of ginger cultivated in Jamaica, viz. the white and the black.

The roots are perennial and digitated. Every fpring they put forth tender fhoots, of which are made the fineft preferves.

Black ginger has the moft numerous and largeft roots, and only requires to be fcalded and dried. The white ginger muft be fcalded in water, and the fkin fcraped off; then carefully dried. This laft bears the beft price.

Ginger is reckoned to impoverifh lands greatly. This, with the trouble and fluctuating ting ftate of the markets, makes only a few people plant it in the mountains.

The virtues and ufes of ginger are well known. In medicine it enters into many compofitions, and merits ftill farther to be employed, as an ufeful fuccedaneum to the more coftly fpices. In Jamaica the common people employ it in baths and fomentations, with good fuccefs, in complaints of the vifcera, in pleurifies, and in obftinate and continued fevers.

Befides the officinal ginger, there are feveral other fpecies of ginger growing wild, differing in fize, flowers, folidity and pungency of the roots, &c. viz.

1. Amomum Zerumbet — *Wild Ginger.*
2. Coftus Arabicus — *Great Wild Ginger.*
3. Alpinia Racemofa — *Mountain Wild Ginger.*

The roots of thefe are whiter, lefs pungent, and fofter than ginger, and are often made into fweetmeats.

4. AMYRIS BALSAMIFERA. — *Rofe Wood.*

This is found on gravelly hills, and rifes to a confiderable height. The trunks are remarkable for having large protuberances on them.

The leaves are laurel ſhaped. The ſmall blue flowers are on a branched ſpike. The berries are ſmall and black.

Roſe wood is an excellent timber: it is replete with a fragrant balſam or oil, and retains its flavour and ſolidity though expoſed to the weather many years.

Perhaps, by ſubjecting this wood to diſtillation, a perfume, equal to the oleum rhodii, may be obtained.

5. ANACARDIUM OCCIDENTALE.—*Caſhew Tree.*

This beautiful and ſhady tree grows to twenty or twenty-five feet high. It bloſſoms early in the ſpring, and continues to flower for ſeveral months. The flowers grow on a branched ſpike: they are ſmall, red, and fragrant.

It is ſomewhat ſingular that the nut or ſeed is firſt produced. It is of a kidney ſhape, and ſoon comes to its natural ſize; which ſo ſoon as it does, the caſhew apple fills up in a few days, being attached to the caſhew nut.

Caſhew apples are red or white; when ripe they are ſoft, and their taſte is agreeably rough and ſweet. Stewed in ſyrup they may be kept many months; and when eaten with milk are highly reſtorative. When the apple is roaſted gently gently and preffed, the juice, with that of lemons or limes, is made into punch.

Betwixt the external covering and the kernel there is a thick brown cauftic oil. This is by fome ufed to take off freckles ; but it inflames fo much, that the remedy is worfe than the difeafe. It appears to be alfo volatile in its effects ; for if cafhew nuts are roafted in a clofe place, the operator's face will be fwelled, inflamed, and covered with a rafh or eryfipelas.

Roafted cafhew nuts are better than chefnuts ; and when blanched in water, and freed from their covering, are as fweet as almonds, and are ufed like them for emulfions.

This tree is of fpeedy growth, as in one year, from the fowing, it bloffoms and bears fruit. The tree lafts many years, and when old, yields a great quantity of tranfparent gum, in no way inferior to gum arabic.

### 6. Andropogon litorale.

I faw this grafs only on the fea fhore, near St. Anne's Bay, Jamaica. It was five feet high, and had jointed ftalks and roots, like the dog grafs of Britain.

A ftrong

A ſtrong decoction of the roots has been ſucceſsfully employed in viſceral obſtructions, given at the rate of three pints a day; but in liver complaints it ſucceeds better, if accompanied by calomel in ſmall doſes.

7. ANNONA MURICATA.—*Sour Sop.*

———-- ſquamoſa.—*Sweet Sop.*

———-- reticulata.—*Cuſtard Apple.*

———-- paluſtris.—*Water,* or *Alligator Apple.*

All theſe grow wild in Jamaica, or are cultivated on account of their fruit.

The ſour ſop is a large fruit, of a heart ſhape, pointed, and beſet with ſpines. When pulled off before maturity, and boiled, it is ſerved at table the ſame as pompions; and if roaſted or baked, is ſimilar to yams. When ripe it is ſoft, ſweet, and deterſive: hence, good in fevers where the mouth is furred.

The ſweet ſop is an agreeable fruit; but the cuſtard apple is eaten only by a few.

The alligator apple grows in rivulets. The root is ſpongy, and as light as cork: it makes excellent ſtrops for razors.

The

The leaves of all ſmell ſtrongly like ſavine, and both they and the fruits are anthelminthic.

8. Arachis Hypogea. — *Ground Nut.*

This is cultivated in gardens, and ſpreads on the ground. It has a yellow-pea bloſſom, and the pods are under the ſurface of the earth, containing two oblong ſeeds.

The toaſted nuts are preferable to cheſnuts. They yield by expreſſion an oil as good as almonds; and when beaten in a wooden or marble mortar, and mixed with water, form an excellent emulſion, not inferior to that of almonds, caſhews, or any other.

9. Argemone Mexicana. — *Yellow Thiſtle.*

This is a common and troubleſome weed. The flowers are yellow; the leaves and ſtems prickly; and, when wounded, a yellow juice runs out, like a ſolution of gum gamboge. The pods are prickly, and contain a number of ſmall black ſeeds; a woman's thimbleful of which are emetic; in a leſſer doſe they are purgative. They are uſed in diarrhœas and dyſenteries.

10. Aristolochia triloba.

———— odoratiſſima.

Both of theſe are called *contrayerva*, and the latter is in common uſe. It grows amongſt the buſhes: its flowers are large and mottled, and cannot fail to attract the notice of the moſt inattentive traveller.

The roots of this ſecond ſpecies are long, equal, and as thick as a man's little finger: they have a ſtrong ſcent, like the radix contrayerva of the ſhops.

The natives of Jamaica uſe a tea or decoction of theſe plants in colds and other febrile complaints; but as the whole genus is acrid and ſtimulating, this often does miſchief; eſpecially where there is an inflammatory diatheſis, or where proper evacuations have not been made.

11. Arum Colocasia. — *White Cocoes.*

———- Sagittæfolium. — *Black Cocoes.*

(*Eddoes* or *Toyos.*)

Theſe two are cultivated as articles of food. The tap root is very large, and ſends out ſhoots or fingers, which, when boiled or roaſted, ſerve inſtead of bread. The parent root is boiled boiled to feed ſwine. The roots yield a great deal of ſtarch.

12. Arum Macrorhizon.—*Cubeſo Wyth.*

This is a climber, and has large round leaves and long wythie roots, from which when cut a white milky reſinous liquor runs out, of a ſtrong turpentine ſmell.

13. Arum divaricatum.—*Paraſitical Cocoes.*

This grows in the boughs of the talleſt trees; the leaves are like thoſe of the cocoes.

The roots both of this and the laſt ſpecies are uſed in decoction, as ſarſaparilla.

14. Arum Arborescens.—*Dumb Cane.*

This grows in moiſt and ſwampy lands, and riſes to ſix or eight feet. Every part of it is acrid. The juice rubbed on the ſkin cauſes an intolerable itching. If eaten through ignorance or deſign, it irritates and even inflames the mouth and fauces, and renders the perſon ſpeechleſs: hence the name.

A phyſician, in the reign of Charles the Second, wrote a treatiſe on the virtues of the dumb cane in dropſy. I have tried it in that diſeaſe, but could not get down a ſufficient quantity to produce the proper effect, on account of its acrimony.

A negro woman, who had been long ailing, in a fit of deſpair, eat a good deal of the dumb cane, with a view to deſtroy herſelf. It excoriated her mouth and throat much, and ſhe voided many worms, but recovered her health ſoon after.

15. Asclepias Curassavica. — *Baſtard Ipecacuanha.* — *The Red Head.*

This is a pretty plant, which grows wild in paſtures. It riſes to three feet; has green ſtems and lanceolated leaves. The flowers ſtand at top in a kind of umbel: they are red and yellow, and very beautiful.

This plant is milky, but not dangerous, like ſome others of this genus. The juice of the leaves is often given to perſons afflicted with worms, from a tea-ſpoonful to an ounce, for a doſe, on an empty ſtomach. In this way I can vouch for its powerful and ſalutary effect. When given in larger doſes, it acts as a mild emetic or purgative; and in worm fevers alſo as a diaphoretic and diuretic. Thus, whilſt it expels worms, it brings about a criſis.

The

The roots are white and woody. When given in powder, as a vomit, they act as an emetic; but this is a dangerous practice.

16. Bixa Orellana. — *Arnotta Bush*, or *Roucou of the Indians.*

This is planted about inclosures, and sometimes rises to twenty feet. The trunks are brown and smooth. The bark is tough, and, by maceration, may be made into a strong hemp or flax.

The flowers are pale red, and very like those of the dog rose. The pods are oval, pointed, and prickly, containing a number of scarlet seeds.

When the pods are ripe they are gathered in baskets; and, when opened, the seeds are thrown into a tub of clean water. The water and seeds are well stirred, and the red adhering substance washed off the seeds; which last are thrown away. The turbid liquor is passed through a hair sieve, and evaporated in a pot over a slow fire to an extract, then made into rolls of a pound weight, which are dried in the shade, and then put up for use.

Arnotta sells at a high price; from fifteen to twenty shillings per pound. It is used as a dye; and in chocolate, to which it communicates a rich and agreeable flavour and tafte as well as colour.

It has been found an ufeful medicine in nephritic and calculous cafes. Half a drachm may be taken in a cup of chocolate twice or three times a day.

The Indians in Spanifh America paint their bodies with arnotta.

17. Bromelia Ananas. — *Pine Apple.*
——— Pinguin. — *Pinguins.*

Pine apples are cultivated in all the Weft-India iflands, and are raifed in every hothoufe in Britain. There are feveral varieties; but the fugar-loaf pine is the beft.

Ripe pine apples are amongft the fineft of our fruits in the Weft Indies, and are relifhed by all ranks of people, efpecially people fick of acute difeafes, dyfenteries, &c. They have a deterfive quality, and are better fitted to cleanfe the mouth and gums than any gargle whatever.

Befides being eaten raw, they are often candied with fugar, and fent home as prefents. Pine apples are alfo made into tarts and pickles.

Pinguins are planted as fences. The fruit is as big as a plum. The juice is exceedingly deterfive, deterſive, and is often employed to clean the mouth. Thin ſlices with ſugar are frequently given to children for worms; but much of it excoriates the mouth and paſſages.

18. BURSERA GUMMIFERA.—*Jamaica Birch.*

This is frequent in woods, and grows ſpeedily to a great height and thickneſs. The bark is brown, and very like the birch of Britain. The wood is ſoft and uſeleſs, except when pieces of the limbs are put into the ground as fences, when it grows readily, and becomes a durable barrier. It has yellow flowers, male and female on different trees. The fruit is a triangular capſule, which, when cut, diſcharges a clear balſam or turpentine.

On wounding the bark, a thick milky liquor is obtained, which ſoon concretes into a reſin no way different from the *gum elemi* of the ſhops.

Dr. Browne, and after him Linnæus, has miſtaken the bark of the roots for the ſimarouba; of which hereafter.

19. CAMO-

19. CAMOCLADIA PUBESCENS. — *Yellow Maſtic.*

This is a fine tall timber tree, frequent in woodlands. The wood is yellow, hard, and takes a fine poliſh.

The whole of this genus is warm or peppery. The bark of the yellow maſtic has an extraordinary taſte, ſomewhat like ardent ſpirits, but more permanent, as, on chewing the ſmalleſt bit, one cannot get the taſte out of the mouth for ſome hours.

The bark retains its pungency when dried, and perhaps may be found an uſeful medicine in lethargic and paralytic diſeaſes, where ſtimulants are indicated.

20. CANELLA ALBA. — *Wild Cinnamon.*

This is a common tree in Jamaica, and grows to a great height. The leaves are oval, ſmooth, and ſhining: the flowers are ſmall, red, and fragrant; they ſtand in form of an umbel, and are ſucceeded by black ſucculent berries, of the ſize of black currants. When ripe, they are ſweet and aromatic: when gathered green, and dried, they are like black pepper, but hotter.

The

The bark is the canella of the ſhops. It enters into various officinal compoſitions, and is a warm, cordial, and aromatic medicine.

The habit and foliage of this tree are very like thoſe of the true Winter's bark. Their ſenſible qualities too are nearly the ſame, and they appear to me to be ſpecies of the ſame genus.

21. Capparis Cynophallophora.—*The Bottle Cod Root.*

This ſhrub is found in copſes, and is diſpoſed to run on buſhes. It is remarkable for having large white flowers, whoſe ſtamina are of an extraordinary length. The pods are a foot long, and unequal. When ripe they open gradually, and ſhew the ſeeds in a ſort of crimſon bedding.

The root is large, yellow, and fleſhy, and taſtes ſtrongly like horſe raddiſh.

Dr. Canvane recommends it as a ſpecific in dropſy. He orders a decoction of it; but an infuſion is preferable, becauſe boiling diſſipates its virtues.

There are ſeveral other ſpecies of Capparis in Jamaica, whose sensible qualities are the same as those of the mustard tribe.

### 22. Capsicum.

Capsicum annuum. — *Cockspur Pepper.*

———-- baccatum. — *Cherry Pepper.*

———-- grossum. — *Gourd Pepper.*

———-- frutescens. — *Bird Pepper.*

———-- ——— (varietas.)—*Hen Pepper.*

———-- galericulum. — *Bonnet Pepper.*

These, and some other varieties, are called Negro Peppers. The bird and hen peppers are indigenous; the others are cultivated in gardens; and all of them have the same sensible qualities, differing only in degrees of pungency. The bird pepper is the smallest, but hotter than any of the others.

All the capsicums may be preserved in vinegar, and form the best of pickles.

When nearly ripe they become red; and if gathered at this time, dried, and powdered, make Cayenne pepper. Some mix common salt; but this is improper, as it disposes the whole to deliquesce, and darkens the colour.

Capsicum has a warm and kindly effect on the ſtomach. It has all the virtues of the oriental ſpices, without producing thoſe complaints of the head which they often occaſion. In food it prevents flatulency from vegetables; but the abuſe of it occaſions viſceral obſtructions, eſpecially of the liver.

In dropſical complaints, or others where chalybeates are indicated, a minute portion of powdered capſicum is an excellent addition.

In lethargic affections this warm and active ſtimulant might be of ſervice. In tropical fevers a coma and delirium are common attendants; and, in ſuch caſes, cataplaſms of capſicum have a ſpeedy and happy effect. They redden the parts, but ſeldom bliſter, unleſs kept on too long.

In ophthalmias, from relaxation of the membranes and coats of the eyes, the diluted juice of capſicum is a ſovereign remedy; and I have often witneſſed its virtues in many obſtinate caſes of this ſort.

In ſome parts of South America the Indians prick the loins and bellies of hectic patients with thorns dipped in the juice of capſicum.

It has been alledged, that capſicum, applied to the loins, would occaſion gonorrhœa. This is contrary to experience, and too ridiculous an opinion to combat feriously.

23. Cassia Occidentalis.—*Pifs a Bed.*

This common weed has a difagreeable fmell, like the leaves of all green caffias. The flowers are yellow; the roots flefhy, and ufed in aperient and diuretic decoctions.

24. Cassia fistula.—*Caffia Tree.*

This tree is cultivated in gardens and about fettlements. It rifes to about thirty feet, and has long flower fpikes, with yellow papilionaceous bloffoms. The pods are about two feet long, and as thick as a man's finger: they are black, fmooth, and fhining. This is the caffia fiftularis of the fhops, and the fame as that brought from the Eaft Indies. The pods of the Caffia Javanica, or horfe caffia, are very large, and the pulp inferior to the former, which enters into fome officinal compofitions.

25. Cassia Senna Italica.—*The round-leaved Senna.*

This grows on fand banks near the fea, particularly ticularly on the palisadoes, near Port Royal in Jamaica.

It rises by herbaceous stems to two feet in height. From the axillæ at the top are sent forth slender spikes, with yellow blossoms. The pods and seeds are the same as those of the senna of the shops. I have dried the leaves, and used them in purging ptisans in the same proportion as those of the Alexandrian senna.

Specimens of this senna were presented to the Society of Arts; and although I received no marks of their approbation, it is with pleasure I observe they have offered a premium lately for raising the Alexandrian senna in the West Indies.

26. CASSIA ALATA. — *Ringworm Bush.*

This plant is annual. The stem is woody, and rises to five or six feet. The leaves are winged, and look like those of walnuts. The flower spikes are simple; the blossoms large, yellow, and placed so closely as to form a cone. The pod is triangular, and four inches long: the seeds are numerous, and heart shaped.

Tetters or ringworms are frequent amongst the black people in Jamaica, and amongst the Spaniards

Spaniards in America very inveterate. I have ſeen this complaint ſo univerſal, that the habit was tainted; the ſkin looked leprous, and the unhappy patient had not a moment's eaſe from the intolerable itching or painful ulcers.

In the beginning a poultice of the flowers of this buſh is of ſervice; as are alſo ſulphureous applications. But, in more advanced ſtages of the diſeaſe, mercurials externally, and the decoction of woods, give the only chance of a cure.

27. CASSIA CHAMÆCRISTA. — *Cane Plant.* — *Senſitive Plant.*

This is frequently met with in cane-piece intervals. It is about three feet in height, and has a few branches, with numerous ſmall pinnated leaves, which collapſe immediately on being touched. The bloſſoms are yellow. The capſule is a flat pod, about an inch long, black, jointed, and ſomewhat hairy. The roots are woody, with many fibres.

In Guinea, and in the Weſt Indies, the negroes are dexterous poiſoners. The plants they employ for this purpoſe are chiefly the lacteſcent ones, ones, of the order *Contortæ*, viz. *Echites suberecta*, *Cameraria*, *Plumeria*, and *Nerium*. An antidote againſt theſe deleterious ſubſtances cannot be too much valued; and ſuch an one is a decoction of the roots of this plant.

A handful of the waſhed roots being boiled in water from three pints to two, may be ſtrained, ſweetened, and uſed for common drink, at the rate of three quarts in twenty-four hours.

28. Cinchona Caribæa.—*Jeſuit's Bark of Jamaica.*

Having given an account of this tree in the Philoſophical Tranſactions, (Vol. lxvii. page 504) with a figure, the reader is referred to that work. I may, however, add, that I have found trees in the pariſh of St. James, in Jamaica, fifty feet high, and proportionally thick. The wood is hard, clouded, and takes a fine poliſh. The bark of the large trunks is rough; the cuticle thick and inert; the inner bark thinner than that of the young trees, but more fibrous.

I have made uſe of this bark in all caſes where the Peruvian bark was indicated, and with the greateſt ſucceſs.

Half

Half an ounce infufed in a bottle of white wine, or fpirits, affords an elegant and grateful bitter. In beginning Typhus I remove the fick into airy chambers, wafh their hands and face often in cold water, and direct them to chew a little of this bark with very happy effects.

29. CINCHONA TRIFLORA.—*The Bath Bark.*

This fpecies of cinchona was difcovered by Mr. Roberts, a clergyman in Jamaica. The leaves are very like thofe of the caribæa. At the axillæ come out three fcarlet flowers. The pods are fomewhat longer than thofe of the laft mentioned fpecies. The bark is of the colour of Peruvian bark. This tree grows only in the parifh of Manchioneel, by rivers.

30. CINCHONA BRACHYCARPA.

Mr. Lindfay, furgeon, and an expert botanift, difcovered this fpecies in the parifh of Weftmoreland, Jamaica, in 1785. It has much the appearance of the following, but very few flowers. It grew on the fide of a fteep hill.

Much has been ſaid and written of late years on the Jeſuit's bark. Sir Joſeph Banks, many years ago, had an elegant plate engraven of the cinchona officinalis, which he diſtributed to his friends. It was by this figure that I was enabled to aſcertain and ſettle the Jeſuit's bark of Jamaica, as well as the other ſpecies I have mentioned.

Of theſe ſpecies, the cinchona Caribæa is the neareſt to the officinal bark in virtue: it abates vomiting, and ſits well on the ſtomach; whereas the other two ſpecies, like the St. Lucia bark, prove emetic in a ſmall doſe. They all, however, cure intermittents.

All theſe different ſpecies are in the poſſeſſion of Sir Joſeph Banks.

32. Cissampelos Pareira.—*Pareira Brava.*

This is a ſlip which runs amongſt the buſhes and on fences. The leaves are round, ſoft, and downy, on which account it is called the velvet leaf.

It bears its flowers on a ſlender pendulous ſpike: they are yellow and very ſmall, and the male and female are on different vines. The fruit is a ſoft, flat berry: it is of a red colour, and contains one flat ſeed curiouſly notched like the wheel of a watch.

The roots are black, ſtringy, and as thick as ſarſaparilla, running ſuperficially under the ſurface of the ground.

This root is agreeably aromatic and bitter, and is recommended by Geoffroy in nephritic diſorders, in ulcers of the kidneys and bladder, in humoral aſthmas, and in ſome ſpecies of jaundice.

The common people in Jamaica uſe a decoction of the roots for pains and weakneſs of the ſtomach proceeding from relaxation.

### 32. Citrus Medica. — *Limes.*
### ——— Limonum. — *Lemons.*

The whole of the genus citrus are natives of Aſia, and the ſouthern parts of Europe, from whence they have been carried to and planted in the warmer parts of America and the ſugar iſlands. At preſent they are ſo common as to be formed into hedges.

The juice of lemons and of limes is nearly alike, and their uſes in medicine and drink well known. About fourteen years ago I wrote a paper paper on the effects of lime juice combined with sea salt in various diseases in the torrid zone*. It is proper to observe, that in all the disorders there mentioned, a remitting fever either occasioned or accompanied them.

In that paper I have slightly mentioned diabetes; but later experience enables me to assert, that in this medicine I have found a specific for diabetes as well as for lienteria, both which diseases have often heretofore baffled the most skilful physicians.

33. CITRUS AURANTIUM DULCIS. — *Sweet Oranges.*
—— —— amara. — *Seville Oranges.*

Both these are cultivated in all the West-India islands, as well as in Spain and Portugal. These ascescent fruits have long been esteemed in medicine, and need not here be insisted on. In the warm countries ulcers soon become very foul and offensive. I have long been of opinion

* Vide American Transactions, Vol. II. and London Medical Journal, Vol. VIII. page 100.

that the habit has nothing to do in many ſuch caſes, but that both the ulcer and the fomes of it are merely local. I have applied the pulp of roaſted oranges to the ſores as a poultice, and obſerved always that in twenty-four hours the fœtor of ſuch ulcers was corrected and removed, and that the ulcers ſoon were diſpoſed to heal. The ſame application was continued till a cure was completed.

### 35. Citrus decumana. — *Shaddock.*

This fruit was ſo called from a Captain Shaddock, who firſt brought it from the Eaſt Indies to Barbadoes.

Shaddocks are a moſt beautiful fruit, about five times as large as oranges, and ſhaped like a pear. They have a moſt agreeably ſweet and bitter taſte, and are much eſteemed in warm countries.

### 36. Citrus decumana, (Varietas.)—*The Forbidden Fruit.*

This is ſmaller than the ſhaddock, and of a round figure. However beautiful to the eye, they are in general ſo bitter and ſour as ſeldom to be eatable.

### 37. Citrus Bergamot.

This is frequent in orchards: it is lefs than an orange, and has a fine fmell.

### 38. Citrus Citrullus. — *Citron.*

This fruit is about double the fize of a lemon, but nearly of the fame fhape. The juice is acid; the fkin remarkably thick.

All the fpecies of citron agree in fome particulars. The leaves and flowers are nearly alike, and on their furface all of them have a volatile fluid, or oil, lodged in fmall round cells, vifible to the naked eye. This effential oil is eafily obtained by diftillation.

The juice of limes, lemons, and oranges, is ufed in fhrub, orangeat, and punch, and enters into many compofitions in pharmacy and confectionary.

The rinds or fkins of citrons, limes, and oranges, make elegant preferves, either in fyrup or candied with fugar.

### 39. Clinopodium rugosum. — *Wild Batchelor's Button.*

This plant is annual, herbaceous, and rifes to three or four feet. The leaves are large, rough, rough, and ferrated; the flowers fmall, and the feed veffels connected in a globular or button-like form.

The leaves of this, beaten and applied to old and obftinate ulcers, have a very good effect. The buttons, when rubbed betwixt the fingers, emit a moft agreeable fragrance, fomewhat like a mixture of the oils of rofemary, lavender, rhodium, and ambergris. As the plant is fo common in all wafte lands, large quantities might eafily be gathered, and this valuable perfume, or oil, obtained by diftillation. The dried pods retain their flavour a confiderable time, and might be fent home in tin canifters or lead cafes to the mother country.

39. Coffea Arabica.—*Coffee Tree.*

It is about fixty years fince coffee was introduced into Jamaica from the Levant. It is now in general cultivation amongft even the meaneft of the people. It flowers twice a year. The bloffoms are white and fweet, like jafmine, and laft a confiderable time. Thefe flowers, with the green fruit, and red ripe berries, on the fame twigs, make a pleafing and beautiful contraft.

The

The fruit is a berry, of the ſize and exact figure of the red cherry. The pulp is ſoft and ſweet, and no doubt might be converted to wine; or, by diſtillation, to brandy. The beans are two in each berry, which are well known.

Coffee is an article of diet, and ſeldom preſcribed in medicine: but I have known it have good effects in the moiſt or humoral aſthma, and to give ſpeedy relief in headachs from gout and other nervous affections. It is ſaid to prevent ſleep; but this happens from any tepid liquors drank late in the evening or at night.

Coffee, with a good deal of milk, is uſed twice a day by moſt families in Jamaica.

40. Convolvulus Brasiliensis.—*Sea-ſide Scammony.*

This plant grows near the ſea ſhore. The leaves are broad and ſhining; the flowers large and pale red.

The roots are thicker than a quill, and run many yards ſuperficially in ſandy places. The whole plant is milky; and if this milk was collected, a reſin, like ſcammony, might be obtained. At preſent this root is employed as a draſtic purge, in dropſy, by the common people.

The Aleppo ſcammony might eaſily be cultivated in Jamaica, and become an uſeful and profitable article. It is growing luxuriantly in His Majeſty's garden at Kew, and in ſeveral other gardens about London.

42. Convolvulus Batatas.— *Sweet Potatoes.*

This ſlip is planted for food, and grows ſo faſt as to be fit to dig up in ſix weeks or two months. For this reaſon, new ſettlers generally plant this as the readieſt proviſion.

The roots have much the appearance of the common potatoe, but are much larger. Theſe, roaſted or boiled, are ſweet, but not ſo farinaceous as the other potatoe, nor do they yield ſo much ſtarch by one half: however, the ſweet potatoe is good ſubſtantial food, and ſerves inſtead of bread, which cannot always be had.

There is a vulgar opinion in Jamaica, that the common or Engliſh potatoe becomes ſweet, and degenerates into this ſlip. The firſt is totally a miſtake; the latter impoſſible.

42. Crescentia Cujete. — *Calabash.*

This useful tree is planted about settlements. The flowers and fruit grow from the body or large limbs of the tree. The fruit, or calabash, is generally large. Some, when hollowed, will contain a gallon of water. The shell serves for utensils for the negroes, as bowls, cups, and spoons.

The contents are white, pretty firm, and contain a number of seeds. The juice of calabash, in the quantity of four ounces, is given as a purge in all cases where the patient has received a bruise about the trunk; and a syrup of the same, with the addition of lime juice, a little nitre, and paregoric elixir, is by some highly extolled in coughs and consumptions.

Small calabashes roasted, and the pulp spread on cloth, make a good poultice for bruises and inflammations.

A smaller calabash grows wild, but is only a mere variety of the other.

43. Croton Eleutheria *.

This tree is common near the sea shore, and rises to about twenty feet. The leaves are from

* *Clutia Eluteria,* Linn.

two to three inches long, and of a proportional breadth. On the upper fide they are waved, and of a rufty colour; on the under fide they are ribbed, and have a fine gloffy or filvery appearance.

From the axillæ they have numerous fmall fpikes, with a great quantity of white, fmall, and fragrant flowers. The capfule is tricoccous, like other crotons.

The bark is the fame as the cafcarilla and eleutheria of the fhops. Medical writers have fuppofed thefe to be diftinct barks, and they are fold in the fhops as different productions; but, when ftrictly examined, they prove to be one and the fame bark.

Linnæus's croton cafcarilla is the wild rofemary fhrub of Jamaica, the bark of which has none of the fenfible qualities of cafcarilla.

44. Daphne lagetto. — *Alligator Bark,* or *Lace Bark Tree.*

Sir Hans Sloane has figured a fprig of this tree, but did not fee the flowers or feeds. Dr. Browne, in his Natural Hiftory of Jamaica, is equally at a lofs with refpect to it; and botanifts were unacquainted with this plant till the year 1777, when I brought complete fpecimens

 of of it from Jamaica, and Sir Jofeph Banks, Dr. Solander, and myfelf, fettled it as a fpecies of Daphne.

The tree grows on the high rocky hills to twenty feet high. The trunks are ftraight; the wood is foft; the bark is thick, and may be feparated into twenty or thirty lamina, white and fine, like gauze. Of this, caps, ruffles, and even whole fuits of ladies' clothes, have been made.

It has the fenfible qualities of mezereon, but in a greater degree. A drachm of it to two pounds of farfaparilla decoction is ufeful in confirmed lues, chronic rheumatifms, and pains of the bones from lues or the yaws.

45. Dioscorea alata. — *Negro Yam.*

——— Bulbifera. — *White Yam.*

——— Sativa. — *Wild Yam.*

——— Triphylla. — *Yampee.*

The two firft fpecies are cultivated in provifion grounds; the flips are climbers, and furnifhed with poles, like hops. They are planted in the fpring, and are ripe about Chriftmas. The roots are very large; fome from thirty to forty pounds weight. They will keep for feveral months, and are in daily ufe as food. Yams,

 roafted roafted or boiled, eat like potatoes, but are rather of a coarfer texture. They are dreffed in various forms, being boiled in foups or broth, &c., made into pudding, or roafted in the fire. They yield alfo a confiderable quantity of ftarch.

The wild yam is a native of the woods in Jamaica. The ftem is angulated, and finely ferrated. If any one lays hold of this vine, it cuts the hands like a knife. The roots are flat, digitated, and large; they are yellow coloured, and very bitter: they purge people unaccuftomed to eat them; but are the chief fupport of the runaway negroes who abfcond from the plantations.

The yampee, till of late years, was little known to the white inhabitants. The leaf is different from the others; the roots are about fix inches long, and two inches in diameter: there are about twelve of fuch to one flip or vine. The Maroons, or mountain negroes, plant them, and bring them down to the low lands. They keep a few weeks. The yampee, boiled or roafted, is a moft delicious root, and far preferable to potatoes.

46. Do-

46. Dolichos pruriens. — *Cowitch.*

This ſlip runs wild amongſt the buſhes in many parts of Jamaica, and now and then is cultivated in gardens.

It is a climber; has ſlender ſtalks; the leaves trifolated; the flowers ſmall and papilionaceous. The pods are about four inches long, round, and as thick as a man's finger, containing a few hard oblong ſeeds.

The outſide of the pods is thickly ſet with ſtiff brown hairs or briſtles, which, when applied to the ſkin, occaſion a moſt intolerable itching.

The ripe pods, when dipped in ſyrup, are ſcraped with a knife, and then thrown away. When the ſyrup, with theſe ſetæ, becomes as thick as honey, it is fit to uſe. It acts mechanically as an anthelminthic; occaſions no uneaſineſs in the firſt paſſages, which are defended by mucus; and may be taken ſafely from a tea-ſpoonful to a table-ſpoonful once a day.

47. Epidendrum Vanilla.

This plant is carefully cultivated in the Spaniſh Weſt Indies, where it is a native. It alſo grows wild in the mountains of Jamaica. Dr.

Swartz,

Swartz, a learned Swedifh botanift, found it there about three years ago.

The pod is a valuable perfume, and fetches a great price. It merits, therefore, the attention of the people, and their reprefentatives in affembly, that it may be cultivated and fent home as an article of commerce.

48. Epidendrum Claviculatum. — *Green Wythe.*

This plant is found on gravelly and rocky lands. It runs or creeps on the ground, taking root here and there in its progrefs. The ftem is as thick as a man's finger, round, green, and fucculent: it is jointed at every twelve or fourteen inches, and is feveral yards long, without leaves. The flowers are large and yellow; the pods two inches long.

On viewing the expreffed juice with a glafs, or the naked eye, we find it full of long fpiculæ or hairs. Dr. Drummond, a learned and ingenious phyfician and botanift in Weftmoreland, Jamaica, who firft fhewed me this plant, affured me, that he had often given a table-fpoonful of the juice as a fafe and effectual vermifuge; and that in fome fpecies of dropfy it promotes a flow of urine, and cures the difeafe. The juice juice is in great efteem, amongft the negroes, for the cure of gonorrhœa and lues venerea.

49. Eupatorium Dalea.

This is frequent in the mountains of Jamaica. It is woody and perennial, and about four feet high. The flowers are yellow; the feeds downy.

The withered ears or leaves, juft dried, have a moft fweet fmell, nearly equal to the vanilla; and we find them often amongft the Spanifh cigaroes, as a perfume, inftead of vanilla.

50. Fevillea Scandens. — *Cacoons.*

This is common in all wafte lands and by the fkirts of the woods. It is a climbing vine, which runs on trees and bufhes for a great way, covering them like ivy.

It has its male flowers on one vine, and the female on another. The bloffoms are fmall and yellow. The fruit is a round calabafh, containing about twelve large flat feeds or nuts. When the fruit is ripe, the feeds fall out at the bottom from a round circular ring or trap door.

The cacoon taftes very bitter, and is oily. The common people employ them as antidotes againft againſt vegetable and fiſh poiſon, as well as in pains and weakneſs of the ſtomach.

The ſeeds, when beaten in a wooden mortar, and boiled long with water, yield an oil or fat, as white and hard as tallow; and they are frequently uſed for this purpoſe at the Muſquito Shore and Honduras, where candles are made of them.

51. GEOFFRÆA INERMIS.—*Cabbage Bark Tree.*

In the ſixty-ſeventh volume of the Philoſophical Tranſactions I have given a botanical and medical account of this tree, to which the Royal Society have added an elegant engraving.

The anthelminthic properties of this bark are pretty generally known; and it is an article of materia medica in the Edinburgh Diſpenſatory, as well as in ſome foreign Diſpenſatories.

Let me in this place remark, that phyſicians expect too much from anthelminthics. The common ſymptoms of worms are often deluſory; for the ſame ſymptoms attend many fevers. When, therefore, the caſe is doubtful, I always join the cinchona officinalis or Caribæa with the cabbage bark.

Worms, expelled in the end of acute diſeaſes, are, in general, a fatal ſymptom; and no no worm medicine ſhould then be given, unleſs the *bark* is given at the ſame time.

52. Abrus Precatorius.—*The Bead Vine.*

This beautiful plant runs on buſhes or fences. It has numerous ſmall and pinnated leaves. The flowers are papilionaceous, and pale red; the pods ſhort and rounded, containing three or four red ſhining ſmall peas, with a black ſpeck at the end.

The leaves and ſtalks are ſweet, and often made into teas or decoctions, to which is added a little lime juice. This drink is uſeful in coughs, colds, and pleuriſies, &c.

The ſeeds are exceedingly hard, and are emetic: they are never eaten or preſcribed. They are common in ſhell ſhops and ſhell works, and are worn as beads by the negroes in Jamaica.

53. Gouana Domingensis.—*Chaw Stick.*

This vine runs wild in fences and in copſes. The ſtalks are woody, flexible, and of the ſize of one's finger: they run to a conſiderable length, and continue of the ſame thickneſs. The leaves are oval, and ſerrated; the flowers

 ſmall

ſmall and white; the capſules ſmall, flat, and white.

Pieces of chaw ſtick are made into tooth bruſhes, and, while they ſerve to clean the teeth, are antiſeptic by their bitterneſs.

This wythe is chewed, and the juice ſwallowed as an agreeable ſtomachic; and is uſeful for promoting an appetite, or removing pains in the ſtomach from relaxation of that viſcus.

What is often called a pain in the ſtomach is an affection of the liver, which ſhould carefully be diſtinguiſhed, as in this caſe all tonics or bitters do miſchief. If the liver is diſeaſed, we have a ſovereign remedy in calomel. One grain for ſix nights running is generally ſufficient.

54. Guajacum officinale.—*Lignum Vitæ.*

This is a native of the Weſt Indies, and grows ſlowly to a middling ſize and thickneſs. Its ſhady ever-green foliage, its numerous azure flowers, and flat yellow pods, make a pleaſing contraſt.

The trunks are commonly crooked; the bark is furrowed, and tears of the gum exude. All the parts of this tree are acrid and diſagreeable to the taſte; and as they contain tain more or leſs reſin, are purgative, diaphoretic, or diuretic.

Beſides the tears found on the trunk, a gum is obtained in the following manner: —The trunk and larger limbs being ſawn into billets of about three feet long, an auger hole is bored lengthways in each, and one end of the billet ſo placed on a fire, that a calabaſh may receive the melted reſin which runs through the hole as the wood burns.

Gum guiacum may be obtained in ſmall quantities by boiling chips, or ſawings, of the wood in water and common ſalt. The reſin ſwims at the top, and may be ſkimmed off.

It may alſo be got by means of ardent ſpirits, in the way Jalap and Peruvian bark are treated; but this mode is expenſive and tedious.

The venereal diſeaſe makes terrible havock amongſt the negroes in Jamaica, and ſhews itſelf in all its hideous forms. This is owing to their ignorance or neglect. Amongſt this claſs of mankind it is too common to ſtop virulent gonorrhœas with aſtringent gums, reſins, or barks, ſo that the maſter or overſeer knows nothing of their ſituation till the ſpongy bones of the noſe, the palate, or the throat, are greatly affected; or their limbs diſtorted by nocturnal pains, pains of the bones, nodes, and carious ulcers.

The yaws, though a very different disease from the lues venerea, often produces the same direful effects in the limbs, nose, and throat: happily, however, these are curable by mercurial alteratives and diaphoretic decoctions.

Of all the preparations of mercury, the corrosive sublimate appears to me to be the best for curing such inveterate disorders, especially when accompanied with such medicines as promote its natural tendency to the skin. Of this sort is guiacum and sarsaparilla. I have found the following formula the best:

Gum guiacum, ten drachms.

Virginia snake root, three drachms.

Pimento, two drachms.

Opium, one drachm.

Corrosive sublimate, half a drachm.

Proof spirits, two pounds.

To be mixed and digested for three days, and then strained.

Two tea-spoonfuls of this tincture given in half a pint of sarsaparilla decoction twice a day, will, in general, remove every symptom of lues or yaws in four or five weeks.

55. Hæ-

55. HÆMATOXYLUM CAMPECHIANUM.—*Logwood.*

Dr. Barham introduced the feeds into Jamaica from Honduras about the year 1715. It is at this time too common, as it has overrun large tracts of land, and is very difficult to root out.

This is generally planted for hedges, and it makes a beautiful and ftrong fence againft cattle or ftock. If pruned from the lower branches, it grows to a fizeable tree, and, when old, the wood is as good as that from Honduras.

The trunks and branches have long, fharp fpines; the leaves are heart fhaped; the flowers, on a fpike, are yellow, tipped with crimfon, fmell fweet, and are exceedingly beautiful. The pod is flat, and contains two or three fmooth long feeds.

Logwood trees are cut up into billets or junks, the bark and white fap of which are chipped off, and the red part, or heart, fent to England for fale.

As a dye, and a medicine, it is well known.

56. HI-

56. HIBISCUS ESCULENTUS.—*Okra.*

This is cultivated in gardens and inclofures as an article of food. It rifes to five or fix feet; has broad leaves, and yellow large flowers. The pod or okra is from two to fix inches long, and one inch diameter. When ripe, it opens longitudinally in five different places, and difcharges a number of heart-fhaped feeds.

The whole of this plant, like others of the *columnifera*, is mucilaginous, efpecially the pods. Thefe are gathered green, cut into pieces, dried, and fent home as prefents, or are boiled in broths or foups for food. It is the chief ingredient in the celebrated pepper pot of the Weft Indies, which is no other than a rich ollá: the other articles are either flefh meat, or dried fifh and capficum. This difh is very palatable and nourifhing.

As a medicine okra is employed in all cafes where emollients and lubricants are indicated.

57. JATROPHA JANIPHA.—*Sweet Caffada.*
———— Manihot.—*Bitter Caffada.*

Both thefe are cultivated as articles of food. It is difficult to diftinguifh the bitter from the fweet caffada by the roots; but it will be beft to to avoid thoſe of the caſſada that bears flowers, as it is the bitter which is poiſonous when raw.

The root of bitter caſſada has no fibrous or woody filaments in the heart of the root, and neither boils nor roaſts ſoft. The ſweet caſſada has all the oppoſite qualities, and is daily ſerved up at table as bread.

Caſſada bread is made of both the bitter and ſweet, thus:—The roots are waſhed and ſcraped clean; then grated into a tub or trough: after this put into a hair bag and ſtrongly preſſed, and the meal or farina dried in a hot ſtone baſon over the fire: laſtly, made into cakes. Theſe make moſt excellent puddings, equal to millet.

The ſcrapings of freſh bitter caſſada are ſucceſsfully applied to ill-diſpoſed ulcers. *[illegible] vol. 6. p. 398.*

Caſſada roots yield a great quantity of ſtarch, which the Brazilians export in little lumps, under the name of *Tapioca*.

58. Jatropha Gossypifolia.—*Belly-ach Buſh.*

——— Curcas. —*Engliſh Phyſic Nut.*

——— Multifida. —*French Phyſic Nut.*

The firſt grows wild; the ſecond is planted round negro gardens; and the third is cultivated as an ornamental ſhrub.

A decoc-

A decoction of the leaves of the two firſt is often uſed with advantage in ſpaſmodic belly-ach, attended with vomiting. It ſits eaſier on the ſtomach than any thing elſe, and ſeldom fails to bring about a diſcharge by ſtool.

The ſeeds of all of them are draſtic purgatives and emetics. They yield, by decoction, an oil of the ſame uſes and virtues as the oleum ricini; of which hereafter.

59. LAETIA APETALA.—*Gum Wood.*

This tree is common in woodlands and copſes: it riſes to a conſiderable height and thickneſs. The trunks are ſmooth and white; the leaves are three inches long, a little ſerrated, and ſomewhat hairy. The ſtamina are yellow, without petals; the fruit is as large as a plum, and, when ripe, opens and ſhews a number of ſmall ſeeds in a reddiſh pulp.

Pieces of the trunk, or branches, ſuſpended in the heat of the ſun, diſcharge a clear turpentine, or balſam, which concretes into a white reſin, and which ſeems to be the ſame as gum ſandarach.

Of this we make pounce: and it appears to me that this turpentine or gum might be uſeful in medicine, like others of the ſame nature.

60. LAN-

60. Lantana Camara. } *Wild Sage.*

——— Aculeata.

——— Involucrata.—*Sea-ſide Sage.*

The firſt grows wild amongſt the buſhes, and is remarkable for the beauty of its flowers, which are yellow, tinged with red.

The ſecond has ſmall white flowers, and dark-coloured rough leaves: it alſo grows wild.

The third ſpecies is found near the ſea. It is a low plant; has ſmall aſh-coloured leaves, and a moſt agreeable ſmell.

The leaves of all theſe lantanas, and particularly of the ſea-ſide ſage, is uſed by the black people in teas, for colds, rheums, and weakneſs of the ſtomach. They are alſo uſed with alum in gargles.

61. Laurus Cinnamomum.—*Cinnamon Tree of Ceylon.*

This noble plant, with other valuable ones, was taken in a French ſhip, and Admiral Rodney, ever attentive to the proſperity of Jamaica, preſented them to the Aſſembly of that iſland.

One of the trees was planted in the botanic garden in St. Thomas in the Eaſt; the other by Hinton Eaſt, Eſq. in his noble garden at the foot of the blue mountains. From theſe parent trees ſome hundreds of young trees are already produced, from layers and cuttings, and diſperſed to different parts of the country, in all which it thrives luxuriantly, with little trouble: we may, therefore, hope it will ſoon be a valuable addition to our commerce.

The ſmalleſt bit of the bark is quite a cordial. The cinnamon we have from Holland is often inert, and gives room to ſuſpect that it has been ſubjected to a ſlight proceſs in diſtillation.

### 62. Laurus Camphora.

This tree is another of the captured plants given to the inhabitants of Jamaica. It is common enough in greenhouſes in Britain.

If cultivated with care, it will alſo be an acquiſition. Camphor, though ſolid, is the eſſential oil of this tree, and is obtained by diſtillation in the Eaſt Indies.

### 63. Laurus Sassafras.

This is a native of North America, and grows luxuriantly in Mr. Eaſt's garden. When propagated, it will alſo be an article of trade from Jamaica.

The roots, and their bark, are uſed in medicine, and the flowers are made into tea, in America, as the raſping of the wood is with us. The ſaſſafras roots and bark are an excellent ingredient in the decoction of woods.

### 64. Laurus Persea. — *Alligator Pear.*

This tree has neither the habit nor ſenſible qualities of the genus Laurus: the flowers, however, have all the generic characters of it.

The alligator pear tree is cultivated univerſally by all ranks of people. It runs ſpeedily to twenty-five or thirty feet in height. The leaves are long, oval, and pointed; the flowers yellow and ſmall. The fruit is pear ſhaped, and from one to two pounds in weight.

On removing a green ſkin or covering, we come to a yellow, butyraceous ſubſtance, and in the heart find a large round ſeed or ſtone. It is unequal in the ſurface, and exceedingly hard and woody.

This fruit is ripe in Auguſt and September, and conſtitutes one of the moſt agreeable articles of diet, for ſix or eight weeks, to the negroes. Theſe pears, with a little ſalt and a plantain or two, afford a hearty meal. They are alſo ſerved up at the tables of white people as choice fruit. When the pear is ripe, the yellow or eatable ſubſtance is firmer than butter, and taſtes ſomewhat like butter or marrow: hence it is called by ſome the vegetable marrow. But however excellent this fruit is when ripe, it is very dangerous when pulled and eaten before maturity. I have repeatedly known it to produce fever and dyſentery, which were removed with difficulty.

The leaves of this tree and thoſe of the bead vine or wild liquorice * are made into pectoral decoctions by the common people.

The large ſtone is uſed for marking linen. The cloth is tied or held over the ſtone, and the letters pricked out by a needle through the cloth and into the ſeed. The ſtain is a reddiſh brown, which never waſhes out.

* Abrus precatorius.

### 65. Malvaceæ (Ordo naturalis.)

Under this title we may comprehend the whole tribe of plants in the ſixteenth claſs of Linnæus, and the natural order *columniferæ*. All of them are mucilaginous, ſaponaceous, and emollient, and may ſafely be employed where mucilaginous and emollient medicines are indicated.

A decoction of the common broomweed * in the Weſt Indies, or of the various ſpecies of Sida, may properly be ſubſtituted in the room of marſhmallow roots.

Many of them yield gums, which are of a ſimilar nature to that of the caſhew. Some are uſed as food, and are highly reſtorative. We ſpoke of this above, under the name of Hibiſcus eſculentus, Okra.

### 66. Maranta Arundinacea.—*Indian Arrow Root.—The Starch Plant.*

This is cultivated in gardens and in proviſion grounds. It riſes to two feet, has broad pointed leaves, ſmall white flowers, and one ſeed.

* Sida alnifolia and rhombifolia.

The

The roots, when a year old, are dug up, well waſhed in water, and then beaten in large deep wooden mortars to a pulp. This is thrown into a large tub of clean water. The whole is then well ſtirred, and the fibrous part wrung out by the hands, and thrown away. The milky liquor being paſſed through a hair ſieve, or coarſe cloth, is ſuffered to ſettle, and the clear water is drained off. At the bottom of the veſſel is a white maſs, which is again mixed with clean water and drained: laſtly, the maſs is dried on ſheets in the ſun, and is pure ſtarch.

A decoction of the freſh roots makes an excellent ptiſan in acute diſeaſes.

67. MIMOSA TORTUOSA. — *Poponax Buſh.*

——— Nilotica. } *Gum-arabic Trees.*

——— Senegal. }

The firſt of theſe has probably been imported, and at preſent grows too abundantly, as it is a thorny troubleſome buſh.

The others have been lately introduced from Guinea. They are trees of about twenty feet high. I ſaw them in the garden of Dr. Paterſon, at Green Iſland, Jamaica. The Nilotica, on on being cut a little, yielded a good deal of tranſparent gum.

Theſe ſeveral ſpecies have ſmall pinnated leaves, which are nearly as ſenſible, on being touched, as thoſe of the mimoſa pudica. The flowers are yellow buttons, which, when rubbed, are very fragrant. All of them afford gum arabic in leſſer or greater quantities, and more or leſs tranſparent.

68. MIRABILIS JALAPA.—*Four o'Clocks.*

This is frequently met with in the gardens of the curious in Great Britain. It grows wild in Jamaica, and is a troubleſome weed. Some have red flowers; ſome yellow; and others, flowers finely variegated.

It has a large tap root, which, when cut acroſs, is not unlike that of Jalap; but when dried is white, light, and ſpongy. It requires to be given in a great quantity to operate as a purge, and is probably the mechoacanna of the ancients, but not the jalap, which belongs to the genus Convolvulus.

69. Musa Paradisiaca.—*Plantain Tree.*
—— Sapientum.—*Banana.*
—— Troglodytarum.—*Wild Plantain.*

The plantain tree is cultivated on a very extenſive ſcale in Jamaica. The fruit is the chief ſupport of the inhabitants.

The leaves are ſix or eight feet long, and from two to three feet broad. The flowers are from a ſpatha, and are covered with purple deciduous calyces. The fruit or plantains are about a foot long, round, and a little bent. When ripe, they grow yellow, ſoft, and ſweet. The ſeeds are larger than muſtard, dark coloured, and numerous: they never vegetate; the tree is propagated by ſhoots.

Plantains are cut when full grown, but before they are ripe. The green ſkin is pulled off, and the heart is roaſted in a clear fire for a few minutes, and frequently turned: it is then ſcraped, and ſerved up as bread. Boiled plantains are not ſo palatable.

The banana tree bears a ſmaller fruit than the plantain. It is never eaten green; but when ripe it is very agreeable, either eaten raw, or fried in ſlices as fritters.

Plantains

Plantains and bananas are eaten by all ranks of people in Jamaica; and but for plantains the iſland would ſcarce be habitable, as no ſpecies of proviſion could ſupply their place. Even flour, or bread itſelf, would be leſs agreeable, and leſs able to ſupport the laborious negro, ſo as to enable him to do his buſineſs or to keep in health.

Plantains alſo fatten horſes, cattle, ſwine, dogs, fowls, and other domeſtic animals.

The wild plantain has no fruit eatable. The leaves of all the ſpecies are nearly alike; and as they are ſmooth and ſoft, they are employed as dreſſings after bliſters.

The water from the ſoft trunk is aſtringent, and employed by ſome to check diarrhœas.

Every other part of the tree is uſeful in different parts of rural œconomy.

70. MYRTUS PIMENTO. — *Allſpice, Jamaica Pepper,* or *Pimento Tree.*

This is a native of Jamaica, and grows in all the woodlands on the north ſide.

Pimento walks are upon a large ſcale, ſince they contain at times ſome hundred acres of ground. This is one of the ſtaple articles of Jamaica.

 This

This tree has bay leaves; the flower resembles that of the elder. The fruit is a black berry as big as a black currant when ripe, and contains two gray smooth seeds.

As soon as the berries are of the proper size, and just before they begin to be ripe, a number of hands are employed to gather them. They are then dried on platforms or sheets, and afterwards put up in bags of one hundred weight for the European market.

Pimento possesses the flavour and qualities of all the eastern spices: it enters into many of the officinal preparations, and is the chief ingredient in the marischal hair powder.

71. PASSIFLORA HEXANGULARIS.—*Granadilla.*
———— Maliformis. — *Water Lemon.*
———— Laurifolia. — *Sweet Calabash.*

All these species are cultivated in Jamaica. They are all eatable; but the pulp of the ripe granadilla is very delicious. Their taste is sweet and subacid, and relished by almost every body, particularly by the sick in acute continued fevers.

The thick rind of unripe granadilla is often made into pickles, or preserved with sugar as sweetmeats.

72. Passiflora rubra. — *The Dutchman's Laudanum.*

This is a ſtrong woody vine that mounts the talleſt trees, and ſends forth vaſt numbers of crimſon flowers. The fruit is black, and of the ſize of a cherry.

A Dutch phyſician, who lived in Hanover pariſh, performed ſome remarkable cures in fevers by the uſe of the flowers and berries; but opium has ſuperior virtues; and the other is now laid aſide as an anodyne of leſs advantage.

73. Picrania amara. — *Bitter Wood.*

This is a tall and beautiful timber tree which is common in the woods of Jamaica. Sir Joſeph Banks had ſprigs of the flowers and ſeeds in ſpirits from me, and we found it a new genus belonging to the *Pentandria Monogynia* of Linnæus. The name is expreſſive of its ſenſible qualities.

Every part of this tree is intenſely bitter; and even after the tree has been laid for floors many years, whoever rubs or ſcrapes the wood feels a great degree of bitterneſs in their mouth or throat. Cabinet work made of this wood is very uſeful, as no inſect will live near it.

This tree has a great affinity to the Quaſſia amara of Linnæus; in lieu of which it is uſed as an antiſeptic in putrid fevers. When uſed, leſs of it will do than of the Quaſſia amara of Surinam *.

74. Piper Amalago.—*Black Pepper of Jamaica.*
——— Inæquale. — *Long Pepper of Ditto.*

Theſe, and ſome other ſpecies, are indigenous, and known by the names of Joint Wood, or Peppery Elders.

The firſt bears a ſmall ſpike, on which are attached a number of ſmall ſeeds of the ſize of

* In 1772 Dr. Wright diſcovered the tree which yields the ſimarouba of the ſhops, and the year following ſent a botanical account of it to the late Profeſſor Hope at Edinburgh, under the title of *Quaſſia Simarouba.* At the ſame time he ſent ſpecimens of it to the late Dr. Fothergill, who tranſmitted them to the celebrated Linnæus at Upſal. The latter communicated this diſcovery to Profeſſor Murray at Gottingen, who has mentioned it in the third volume of his *Apparatus Med.* page 458.

We are happy to learn from Dr. Wright, that he means ſoon to publiſh a deſcription of this tree, accompanied with engravings.

 muſtard.

muftard. The whole of the plant has the exact tafte of the Eaft-India black pepper.

The long-pepper bufh grows taller than the amalago. The leaves are broad, fmooth, and fhining. The fruit is fimilar to the long pepper of the fhops, but fmaller.

The common people in Jamaica feafon their meffes with the black pepper.

To preferve both, the fruit may be flightly fcalded when green, then dried, and wrapped in paper. Perhaps hereafter they may be deemed worthy of attention.

### 75. Portlandia Grandiflora.

Dr. Browne has defcribed this plant, and given a good figure of it. It has frequently flowered in the King's garden at Kew, and in Dr. Pitcairn's at Iflington.

The external bark is remarkably rough, furrowed, and thick: it has no tafte. The inner bark is very thin, and of a dark brown colour. Its tafte is bitter and aftringent, and its virtues are the fame as thofe of the Jefuit's bark. Infufed in fpirits, or wine, with a little orange peel, it makes an excellent ftomachic tincture.

76. Ricinus Communis.—*Palma Christi.*—*Castor-oil Nut Tree.*

This tree is of speedy growth, as in one year it arrives at its full height, which seldom exceeds twenty feet. The trunk is subligneous; the pith is large; the leaves broad and palmated; the flower spike is simple, and thickly set with yellow blossoms in the shape of a cone; the capsules are triangular and prickly, containing three smooth gray mottled seeds.

When the bunches begin to turn black, they are gathered, dried in the sun, and the seeds picked out. They are afterwards put up for use as wanted, or for exportation.

Castor oil is obtained either by expression or by decoction. The first method is practised in England; the latter in Jamaica. It is common first to parch the nuts or seeds in an iron pot over the fire; but this gives the oil an empyreumatic taste, smell, and colour: and it is best prepared in this manner:—

A large iron pot or boiler is first prepared, and half filled with water. The nuts are then beaten in parcels in deep wooden mortars, and, after after a quantity is beaten, it is thrown into the iron veſſel. The fire is then lighted, and the liquor is gently boiled for two hours, and kept conſtantly ſtirred. About this time the oil begins to ſeparate, and ſwims on the top, mixed with a white froth, and is ſkimmed off till no more riſes. The ſkimmings are heated in a ſmall iron pot, and ſtrained through a cloth. When cold, it is put up in jars or bottles for uſe.

Caſtor oil, thus made, is clear and well flavoured, and, if put into proper bottles, will keep ſweet for years.

The expreſſed caſtor oil ſoon turns rancid, becauſe the mucilaginous and acrid parts of the nut are ſqueezed out with the oil. On this account I give the preference to well prepared oil by decoction.

An Engliſh gallon of the ſeeds yield about two pounds of oil, which is a great proportion.

Before the diſturbances in America, the planters imported train oil for lamps and other purpoſes about ſugar works. It is now found that the caſtor oil can be procured as cheap as the fiſh oil of America: it burns clearer, and has not any offenſive ſmell. This oil, too, is fit for all all the purpofes of the painter, or for the apothecary in ointments and plafters.

As a medicine, it purges without ftimulus, and is fo mild as to be given to infants foon after birth, to purge off the meconium. All oils are noxious to infects, but the caftor oil kills and expels them. It is generally given as a purge after ufing the cabbage bark fome days.

In conftipation and belly-ach this oil is ufed with remarkable fuccefs. It fits well on the ftomach, allays the fpafm, and brings about a plentiful evacuation by ftool, efpecially if at the fame time fomentations, or the warm bath, are ufed.

Belly-ach is at prefent lefs frequent in Jamaica than formerly, owing to feveral caufes. The inhabitants, in general, live better, and drink better liquors; but the exceffive drinking of new rum ftill makes it frequent amongft foldiers, failors, and the lower order of white people. I have known it happen too from vifceral obftructions after intermittents, or marfh fevers, in Jamaica.

77. Saccharum officinale.—*Sugar Cane.*

This is a native of Africa, the Eaſt Indies, and of Brazil; from whence it was introduced into our Weſt-India iſlands ſoon after they were ſettled.

The ſugar cane is the glory and the pride of thoſe iſlands. It amply rewards the induſtrious planter, enriches the Britiſh merchant, gives bread to thouſands of manufacturers and ſeamen, and brings an immenſe revenue to the Crown.

It is not meant here to ſay any thing of the proceſs for making ſugar. This has been done by ſeveral hands, and particularly by Colonel Martin, of Antigua, and by Dr. Grainger, late of St. Chriſtopher's, in his elegant poem of the Sugar Cane.

Sugar, formerly a luxury, is now become one of the neceſſaries of life. In crop time every negro on the plantations, and every animal, even the dogs, grow fat. This ſufficiently points out the nouriſhing and healthy qualities of ſugar. It has been alledged that the eating of ſugar ſpoils the colour of, and corrupts, the teeth: this, however, proves to be a miſtake, for no people on the earth have finer teeth than the negroes in Jamaica.

Dr. Alfton, formerly profeffor of botany and materia medica at Edinburgh, endeavoured to obviate this vulgar opinion: he had a fine fet of teeth, which he afcribed folely to his eating great quantities of fugar.

In medicine I need fay little of the ufe of fugar. Externally it is often ufeful: mixed with the pulp of roafted oranges *, and applied to putrid or ill-difpofed ulcers, it proves a powerful corrector.

78. Sesamum indicum.—*Vanglo.*

The oil feed, or vanglo plant, was firft introduced into Jamaica by the Jews as an article of food. It is cultivated in gardens and provifion grounds.

The plant is annual and herbaceous, rifing to about three feet. The flowers are numerous, white, and belong to the clafs *Didynamia* of Linnæus. The pods are about the thicknefs of one's little finger, and contain a great number of fmall white feeds.

In diet the negroes boil this in foups and broths, inftead of flefh meat. The Jews, befides this, make cakes of it to eat as bread.

* Vide Citrus.

The expreſſed oil is as clear and ſweet as oil of almonds, and keeps better. The Behen's oil, ſo uſeful for the fineſt varniſh in coach painting, is probably no other than that of the vanglo. The proportion of oil in this ſeed is great, nine pounds yielding two pounds of oil.

79. Smilax Sarsaparilla.—*Sarſaparilla Root.*

Several ſpecies of ſmilax have roots nearly ſimilar; but that from Honduras and Campechy is the beſt.

This ſpecies has ſtems of the thickneſs of a man's finger: they are jointed, triangular, and beſet with crooked ſpines. The leaves are alternate; ſmooth and ſhining on the upper ſide; on the other ſide are three nerves, or coſtæ, with ſundry ſmall crooked ſpines. The flower is yellow, mixed with red. The fruit is a black berry, containing ſeveral brown ſeeds.

Sarſaparilla delights in low, moiſt grounds, and near the banks of rivers. The roots run ſuperficially under the ſurface of the ground. The gatherers have only to looſen the ſoil a little, and to draw out the long fibres with a wooden hook. In this manner they proceed till the whole root is got out. It is then cleared of the mud, dried, and made into bundles.

The ſenſible qualities of ſarſaparilla are mucilaginous and farinaceous, with a ſlight degree of acrimony. The latter, however, is ſo ſlight as not to be perceived by many; and I am apt to believe that its medicinal powers may fairly be aſcribed to its demulcent and farinaceous qualities.

Since the publication of Sir William Fordyce's paper on ſarſaparilla in the Medical Obſervations and Inquiries, Volume the Firſt, ſarſaparilla has been in more general uſe than formerly. The planters in Jamaica ſupply their eſtates with great quantities of it; and its exhibition has been attended with very happy conſequences in the yaws and in venereal affections, as nodes, tophi, and exoſtoſis, pains of the bones, and carious or cancerous ulcers.

Sir William Fordyce ſeems to think ſarſaparilla a ſpecific in all ſtages of lues; but from an attentive and careful obſervation of its effects in ſome thouſands of caſes, I muſt declare I could place no dependence on ſarſaparilla alone. But if mercury had formerly been tried, or was uſed along with ſarſaparilla, a ſpeedy cure was ſoon effected. Where the patients had been reduced by pain, diſorder, and mercury, I preſcribed a decoction of ſarſaparilla, and a table-ſpoonful of the powder of the ſame, twice a day, with the greateſt ſucceſs, in the moſt deplorable caſes of lues, ill-cured yaws, and carious or ill-diſpoſed ſores, or cancers.

There are only a few ſarſaparilla plants in Jamaica; but it might be cultivated there, and ſave the planter an immenſe expence.

We have alſo the China root growing wild in Jamaica; but it is ſeldom uſed in practice.

80. SPIGELIA ANTHELMINTHICA.—*Worm Graſs.*

Worm graſs grows wild in ſome parts of Jamaica, but is commonly planted in gardens. It grows ſometimes to two feet in height. Dr. Browne gives a very juſt figure of this plant.

The flowers are ſmall and white; the capſules are round, and contain a great quantity of ſmall ſeeds.

Worm graſs has long been in repute as a vermifuge, and is in daily uſe as ſuch in Jamaica. Its action is ſimilar to that of the ſpigelia marilandica. Moſt vegetable anthelminthics have leſs or more of a narcotic effect; and this genus, in a full doſe, brightens the coats of the eyes and diſtends their veſſels: it alſo occaſions ſleep, and hence is uſeful in worm fever.

After its uſe for ſome days, a doſe of caſtor oil is generally ordered. Let me here again be permitted to repeat the uncertainty of the ſigns of worms, eſpecially in fever, and to caution the public againſt depending on anthelminthics alone in their cure. The Jeſuit's bark ſhould be given in all doubtful caſes, or where worm medicines fail in their effects.

81. Swietenia Mahagoni.—*The Mahogony Tree of Jamaica.*

This tree grows to a moſt majeſtic ſize and height. It is of ſlow growth and great hardneſs. The wood is well known in Britain.

Mahogony was formerly very plentiful in Jamaica, but is now only in the high hills, and difficult of acceſs.

The trunk is generally ſtraight; the bark rough, ſcaly, and brown; that on the boughs and twigs is gray and ſmoother. The bark of theſe laſt, dried, is very like the Peruvian bark in colour as well as in taſte, but has more bitterneſs.

Mahogony bark, infuſed in wine or ſpirits, makes an elegant tincture, which reſembles the tincture of the beſt Jeſuit's bark, for which it is often ſubſtituted; and I have ſeen the powder powder adminiftered in intermittents with fuccefs when the Peruvian bark could not be had.

82. Tamarindus Indica. — *The Tamarind Tree.*

This beautiful, fhady, and ufeful tree is cultivated all over the Weft Indies. It rifes to thirty or forty feet high. The trunk is brown, fcaly, and of a good fize. The wood is brown, very hard, and takes a fine polifh.

The branches are fpreading: the leaves fmall, numerous, and pinnated. The flowers yellow, and beautifully ftreaked with crimfon; they continue during the whole of June and July, and then drop off.

The fruit is a broad, afh-coloured pod. The external covering is thin and brittle. This being removed, we find feveral hard feeds, like beans, enveloped in a foft brown pulp, which is fecured by fundry longitudinal woody fibres. This fruit is ripe about Eafter, when it is picked off the trees, and put up for ufe.

Tamarinds are prepared or cured two ways. The common way is to throw hot fugar from the boilers on the ripe pulp: but a better method is to put alternate layers of tamarinds and powdered ſugar in a ſtone jar. By this means the tamarinds preſerve their colour, and taſte more agreeably. The ſeeds, too, of tamarinds, thus prepared, will vegetate eaſily; and this method conveys a hint for ſending ſucculent berries and ſeeds in tamarinds from abroad.

Preſerved tamarinds are kept in moſt houſes in Jamaica, either as a ſweetmeat, or for occaſional uſe as a medicine. They are cooling, laxative, and antiſeptic: hence uſeful in acute and putrid diſeaſes.

Dr. Zimmerman preſcribes tamarinds in putrid dyſentery. I commonly add a portion of Epſom ſalts till ſtools are procured; afterwards, tamarinds alone till the diſorder is cured.

In obſtinate dyſenteries I have found five grains of calomel act like a charm, whether the diſorder was kept up by bilious obſtructions or worms.

83. THEOBROMA CACAO.—*Chocolate Tree.*

In all the French and Spaniſh iſlands and ſettlements, in the warmer parts of America, the chocolate tree is carefully cultivated. This was was formerly the cafe alfo in Jamaica; but at prefent we have only a few ftraggling trees left, as monuments of our indolence and bad policy.

This tree delights in fhady places and deep valleys. It is feldom above twenty feet high. The leaves are oblong, large, and pointed. The flowers fpring from the trunk and large branches; they are fmall, and pale red. The pods are oval and pointed. The feeds, or nuts, are numerous, and curioufly ftowed in a white pithy fubftance.

The cocoa nuts being gently parched in an iron pot over the fire, the external covering feparates eafily. The kernel is levigated on a fmooth ftone; a little arnotto is added, and, with a few drops of water, is reduced to a mafs, and formed into rolls of one pound each. This fimple preparation is the moft natural, and the beft. It is in daily ufe in moft families in Jamaica, and feems well adapted for rearing of children.

84. Verbena Jamaicensis.—*Vervain.*

This is a common weed about all cultivated places. The leaves are ferrated, and pretty broad; the flowers blue.

 A tea,

A tea, or a ſtrong decoction, of vervain is in frequent uſe as a cooling laxative; and a tea-cupful of the expreſſed juice of bruiſed vervain leaves is a ſmart purge.

85. ZANTHOXYLUM CLAVA HERCULIS.
———————— trifoliatum.

The firſt of theſe is the prickly yellow wood, and is a lofty and good timber tree. The ſecond is called the tooth-ach tree. It is frequent in gravelly places near the ſea.

The berries of both are ſomewhat peppery, and a bit of the bark from the roots is a powerful ſialogogue, and gives that ſort of ſenſation as if the mouth was full of blood: hence it is ſo ſerviceable in tooth-ach.

86. ZEA MAYS.—*Indian Corn*, or *Mayz*.

Indian corn, or mayz, is cultivatd in America as an article of food; as it is alſo in Jamaica. The mayz of North America is white, flat, ſpongy, and of the ſize of a dried Turkey bean. The mayz of Jamaica is much ſmaller, reddiſh, and compact. The grains are faſtened to a light ſpongy ſubſtance, called the huſk, or corn ſtick, in longitudinal rows, about twelve in number, round, and containing thirty grains in each. For the moſt part, there are two or three ſuch heads on every ſtalk. The increaſe is prodigious.

Guinea corn, or Indian millet, is alſo cultivated to a great extent in Jamaica. Theſe corns do not conſtitute a great part of the ſupport of the inhabitants of Jamaica, but are chiefly uſed to rear poultry, to feed horſes, and to fatten pigs, goats, or ſheep.

### PALMÆ.

Of this natural order we have ſeveral in Jamaica; ſome of which are indigenous; others have been introduced.

87. COCOS NUCIFERA.—*Cocoa Nuts.*

———— Guineenſis.—*Prickly Pole.*

The cocoa-nut tree was originally brought from the Spaniſh main to Jamaica, and is now planted about ſettlements as an uſeful and ornamental tree. It bears fruit about ten or twelve years after it is planted. The fruit is large, triangular, about twelve inches long, and nine inches in diameter. After removing the external covering and a fibrous ſubſtance, we find a large, round, hard nut, in which is contained about eight ounces of ſweetiſh water, ſurrounded by a white and firm kernel.

The rib of the leaves or pinnæ is ſmooth and flexible, and is uſed in the heart of bougies. The leaves and their ſtems are uſeful for thatching houſes, or making baſkets. The curious reticular cloth, which covers the tender foot ſtalks, ſerves for ſtrainers. A liquor drawn from the trunk, fermented with rice, makes arrack. The fibrous ſubſtance covering the nut, ſpun and twiſted, makes ſtrong and durable ropes. The ſhell is converted into drinking cups, ſugar diſhes, &c. The water is pleaſant, and uſed to quench thirſt. Before the fruit is quite ripe the nut is ſoft, and may be eaten with a ſpoon; but when ripe it is hard. Like other nuts, it is apt to give a pain in the ſtomach. A ſort of tarts, or cheeſecakes, is made from the dry nut kernels, raſped or pared down. This may alſo be uſed for emulſions, inſtead of almonds; and, by expreſſion or decoction, theſe kernels yield a conſiderable quantity of oil.

The prickly pole is a native of low and upland valleys: it riſes to about thirty feet. The trunk and leaves are beſet with ſpines in form of needles. The fruit is of the ſize of hiccory nuts, nuts, and very hard. The black people boil the nuts in their meſſes; and if boiled in water, a yellow thick oil, or butter, is obtained.

88. Cocos butyracea.—*The Mackaw Tree.*

This was originally brought from Guinea by the negroes. The trunk is ſtraight, and guarded by numerous long ſpines, or needles. The fruit is triangular, yellow, and as big as a plum. The nut, or kernel, by decoction, yields the oleum palmæ of the ſhops.

The fruit of this and the former ſerve to feed ſwine, and are greedily eaten by the wild hog, of which there are ſtill many in the interior parts of the iſland.

89. Areca Oleracea—*Cabbage Tree.*

This is a native of the woods. The trunk is ſtraight, and marked with rings at the veſtigiæ of the foot ſtalks of the leaves. Theſe leaves ſpread out at the ſummit in form of an umbrella, and are about three yards in length, and pinnated. The foot ſtalks at the bottom are broad, and form a green trunk above the woody or true ſummit. As the lower leaves drop, the broad part of the foot ſtalks forms a hollow hollow trough, or cradle for negro children; and when cut up makes excellent ſplints for fractures. On the inner ſide of every tender foot ſtalk are tender pellicles, which, when dried, make a writing paper. The heart is made into pickles, or, when boiled, is ſerved up at table. The trunks ſerve as gutterings; the pith makes a ſort of ſago; and the nuts yield oil by decoction.

Of all trees in the univerſe, this is the moſt beautiful, and perhaps the talleſt. I have ſeen one an hundred and ſeventy feet high, and have heard of others ſtill taller.

### 90. *The Sago Palm.*

This valuable palm tree was preſented to the iſland by Admiral Rodney, with many other valuable plants captured in a French ſhip by Captain Marſhall.

This plant was but young when I ſaw it; but as it was healthy, and carefully attended to in Mr. Eaſt's garden, it is hoped it will thrive, and in time be propagated by the ſeeds.

In Amboyna, and ſeveral other parts of the Eaſt Indies, ſago is made from this tree.

The

The pith is beaten into a ſtiff paſte; then granulated through a ſieve in the ſame manner as the grains of gunpowder are formed.

The *ſago powder* ſold in the ſhops is merely the ſtarch of potatoes; and the *tapioca* of the Brazils is the ſtarch of caſſada.

See the articles Jatropha and Maranta.

91. PHOENIX DACTYLIFERA.—*Date Tree.*

This tree is not indigenous, but was introduced ſoon after the conqueſt of the iſland by the Spaniards. There are, however, but few of them in Jamaica at this time. The fruit is ſerved up as a deſert; and the kernels yield an oil, or butter, ſimilar to the palm oil from Guinea.

There are ſeveral other palms growing wild in Jamaica, viz. the mountain thatch, the palmeto thatch, the palmeto royal, &c. The fruit is either a drupa, or a berry, and all of them have one or more nuts, which contain a kernel that yields oil. This circumſtance, with the great reſemblance in their habit, makes them truly a natural claſs, or family.

