# Errata

**Published:** 1787

**Authors:** 


					F. 238, for Cane riant?Serfillvc Plant, read
Cine Piece Senfitivc. Plant.
the end.

				

